# Field evaluation of anticoccidial efficacy: A novel approach demonstrates reduced efficacy of toltrazuril against ovine *Eimeria* spp. in Norway

**DOI:** 10.1016/j.ijpddr.2018.05.002

**Published:** 2018-05-16

**Authors:** Ane Odden, Matthew J. Denwood, Snorre Stuen, Lucy J. Robertson, Antonio Ruiz, Inger Sofie Hamnes, Lisbeth Hektoen, Heidi L. Enemark

**Affiliations:** a Norwegian University of Life Sciences, Faculty of Veterinary Medicine, Department of Production Animal Clinical Sciences, Kyrkjevegen 332/334, 4325 Sandnes, Norway; b Department of Veterinary and Animal Sciences, Faculty of Health and Medical Sciences, University of Copenhagen, Frederiksberg, Denmark; c Norwegian University of Life Sciences, Faculty of Veterinary Medicine, Department of Food Safety and Infection Biology, P.O. Box 369 Sentrum, 0102 Oslo, Norway; d Parasitology Unit, Department of Animal Pathology, Faculty of Veterinary Medicine, University of Las Palmas de Gran Canaria, 35416 Arucas, Las Palmas, Spain; e Norwegian Veterinary Institute, Ullevålsveien 68, P.O. Box 750 Sentrum, 0106 Oslo, Norway; f Animalia, Norwegian Meat and Poultry Research Centre, P.O. Box 396, Økern, 0513 Oslo, Norway

**Keywords:** *Eimeria* spp., Anticoccidial efficacy, Drug resistance, Field evaluation, Norway, Sheep

## Abstract

Ovine *Eimeria* spp. infections cause reduced welfare, increased mortality, and substantial economic losses, and anticoccidials are crucial for their control. Recent reports of toltrazuril resistance in pigs, and anecdotal reports of reduced anticoccidial efficacy in lambs, necessitate evaluation of anticoccidial efficacy. Due to the substantial lifecycle differences between nematodes and coccidia, current WAAVP methods for assessing anthelmintic efficacy are not suitable for such evaluations. Faecal samples were collected from 8 pairs of twin lambs from 36 Norwegian sheep farms 6–8 days after turnout. One twin of each pair was then treated with 20 mg/kg toltrazuril and a second faecal sample from all lambs was collected 7–11 days later. Oocyst excretion rate in all samples was determined using McMasters. Suitability of treatment timing was investigated by evaluating the increase in mean log oocyst excretion in untreated lambs. Based on comparisons between groups, a threshold of ≥0.75 (13 farms) was used to identify farms where drug efficacy could be assessed with confidence, drug efficacy on farms with increases of ≥0.5 but <0.75 (7 farms) were evaluated with caution, and drug efficacy on farms with increases of <0.5 (16 farms) was not estimated. Reduction in oocyst excretion between samples from treated lambs compared with controls from the 20 farms with a threshold of ≥0.5 were then analysed using a generalised linear mixed model. The results were classified based on 95% CI obtained using parametric bootstrapping. Among these 20 farms, two exhibited reduced drug efficacy (upper 95% CI < 95%), 13 had good efficacy (lower 95% CI > 90%), and for 5 the results were inconclusive. This is the first evidence-based report of reduced anticoccidial efficacy in ovine *Eimeria* spp. Additionally, we highlight the problem of sub-optimal timing of treatment (16/36 farms), which could potentially result in incorrect conclusions being reached regarding lack of drug efficacy.

## Highlights

• Field evaluation of anticoccidial efficacy in twin lambs based on oocyst counts.
• Heuristic using evaluation of control animals to ensure a robust FOCR estimate.
• First evidence of reduced toltrazuril efficacy against ovine *Eimeria* spp.
• Sub-optimal timing of treatment was found in 16 of the 36 study farms.
• Drug efficacy was reduced in 2 of 13 farms with appropriate timing of treatment.

## Graphical abstract

**Figure:**
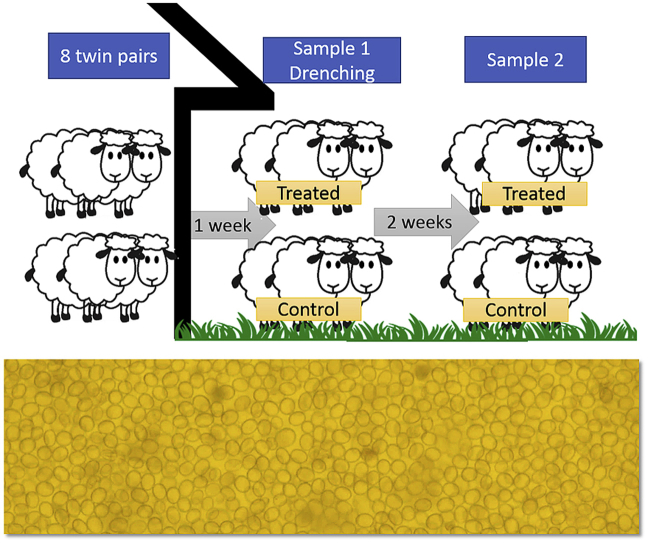

## 1 Introduction

*Eimeria* spp. are host-specific obligate intracellular protozoan parasites that infect fish, reptiles, birds and mammals (Walker et al., 2013). Of the 15 *Eimeria* spp. known to infect sheep, only two are regarded as major pathogens: *E. ovinoidalis* and *E. crandallis* (Catchpole et al., 1976; Catchpole and Gregory, 1985; Rommel, 2000; Joachim et al., 2018). *E. ahsata,* and occasionally *E. bakuensis,* are generally considered to be minor pathogens, which may cause clinical signs in heavily infected animals (Mahrt and Sherrick, 1965; Deplazes et al., 2016). In addition, infections with multiple species might also be important for the development of clinical signs, as described for calves (Enemark et al., 2013). Coccidiosis in lambs caused by pathogenic *Eimeria* spp. leads to reduced welfare, increased mortality and substantial economic losses in the sheep industry worldwide (Foreyt, 1990; Chartier and Paraud, 2012).

Pasture management and hygienic measures, e.g., cleaning water troughs and maintaining dry bedding, are considered important factors for reducing the infection pressure from *Eimeria* spp. (Taylor, 2000; Daugschies and Najdrowski, 2005). However, these measures are often labour intensive and can be difficult to implement, and chemoprophylaxis with anticoccidials is therefore frequently used, in addition to hygiene measures for control of clinical coccidiosis in sheep farms (Taylor and Kenny, 1988; Platzer et al., 2005; Saratsis et al., 2013; Odden et al., 2017). Metaphylactic administration of a single oral treatment with toltrazuril in the prepatent period has been shown to be effective at reducing clinical signs and maintaining adequate growth rates in different production systems (Gjerde and Helle, 1986, 1991; Taylor and Kenny, 1988; Le Sueur et al., 2009; Saratsis et al., 2013). In several European countries (e.g., Denmark, Sweden, and Norway) toltrazuril is the only anticoccidial available for use in sheep (Felleskatalogen, 2017; Läkemedelsverket, 2017; Veterinærmedicinsk Industriforening, 2017). In other countries, other treatments such as diclazuril and decoquinate are also available (Taylor, 2000; Diaferia et al., 2013). According to the Veterinary Medicines Directorate, 510,388 kg and 787,300 kg toltrazuril from products authorised for use in farm animals were sold in the UK in 2014 and 2015, respectively (Dr Gillian Diesel, Head of the Pharmacovigilance Team, personal communication). However, treatment of clinical coccidiosis is considered to be inefficient due to the extensive intestinal damage caused by the parasite (Mundt et al., 2003; Taylor et al., 2003).

Anticoccidial resistance (ACR) is a widely recognised problem in poultry production (Chapman, 1997; Stephan et al., 1997; Chapman and Jeffers, 2014; Lan et al., 2017), and has been reported for monensin, salinomycin, nicarbazin, halofuginone, robenidine, toltrazuril and diclazuril (McDougald, 1981; Chapman, 1997; Stephan et al., 1997). ACR in poultry production is generally considered to be the result of intensive use of anticoccidials, which has led to loss of sensitivity to these drugs (Peek and Landman, 2011). Testing for anticoccidial efficacy (ACE) in poultry production involves the use of histopathological observations and the combination of different indexes, such as oocyst index, body weight gain, relative weight gain, lesion scores, and anticoccidial index (Chapman, 1998). However, no such methods have been published for the evaluation of anticoccidial efficacy in other livestock, including sheep. One obvious practical requirement for a method to be useful in field situations is that it should not include euthanasia of large numbers of animals.

The controlled efficacy test (CET) is the gold standard method for the evaluation of anthelmintic efficacy (Wood et al., 1995; Coles et al., 2006). The CET is performed by infecting animals with a suspected resistant isolate, treating the animals with the drug under evaluation, and then euthanizing the animals before quantifying the parasite burden post mortem. This procedure has various difficulties for implementation, not only because of the ethical concerns associated with euthanasia of the animals, but also due to the requirement for a parasite-free environment in testing the suspected strain (Taylor et al., 1995; Wood et al., 1995). Similar problems relate to the assessment of anticoccidial efficacy against *Eimeria* spp. in poultry (Chapman, 1998) and *Cytosisospora suis* in pigs (Shrestha et al., 2017). Thus, evaluation of anthelmintic efficacy in animals is routinely assessed by the faecal egg count reduction test (FECRT), currently recommended by the World Association for the Advancement of Veterinary Parasitology (WAAVP), and involves comparison of faecal egg counts pre- and post-treatment (Coles et al., 1992). The advantage of the FECRT is its ability to assess a range of drugs under field conditions. However, analysis of the results for FECRTs can be difficult in cases where egg excretion rate is low, where the sensitivity of the counting methods is poor, for highly aggregated faecal egg counts, and when the sample size is small (Torgerson et al., 2005; Denwood et al., 2010; Dobson et al., 2012; Peña-Espinoza et al., 2014).

Different statistical models have been applied to improve the calculation of the estimated efficacy from FECRT results, including bootstrapping techniques, and Bayesian methods such as Markov chain Monte Carlo (Denwood et al., 2010; Torgerson et al., 2014; Peña-Espinoza et al., 2016). However, challenges remain regarding the use of faecal oocyst count reduction tests (FOCRT), the (coccidial) oocyst equivalent of FECRT, for the assessment of ACE, due to extreme variation in oocyst excretion rates compared with excretion of helminth eggs. This is, in general, a reflection of the more complicated biology and lifecycle of *Eimeria* spp., in which sexual reproduction of the parasite in the animal host is preceded by several rounds of intracellular asexual reproduction that occurs in waves (Walker et al., 2013). The maximum range of *Eimeria* oocyst excretion can differ from between 0 and 75,000 to between 0 and 2,000,000 oocyst per gram (OPG), with large inter-individual variation (Chapman, 1974). In contrast, helminth egg excretion usually does not exceed 20,000 eggs per gram (Sréter et al., 1994; Zaros et al., 2014). As toltrazuril acts against intracellular stages of the parasite, and extracellular stages are unaffected (Haberkorn and Stoltefuss, 1987; Harder and Haberkorn, 1989; Mehlhorn, 2008), oocyst counts immediately post treatment may not be zero. Detailed data concerning the efficacy of toltrazuril when the drug was first marketed are not available from the literature, but practical experience confirms that post-treatment oocyst counts are not always zero even when the observed clinical efficacy is good. Thus, any model for evaluation of ACE has to take into account that a reduction to zero is not always the case, even when highly efficacious anticoccidials are used (Taylor and Kenny, 1988; Gjerde and Helle, 1991).

The emergence of ACR in poultry and pig production systems (Lan et al., 2017; Shrestha et al., 2017), along with anecdotal reports of reduced ACE in Norwegian lambs (Odden et al., 2017), demonstrates the need for a FECRT-type method to evaluate drug efficacy in live animals. However, due to the reasons outlined above, the standard FECRT (Coles et al., 1992) currently recommended by WAAVP for evaluation of anthelmintic efficacy is unsuitable for use with coccidia. The aim of our study was therefore to develop a tool for field evaluation of ACE, based on oocyst counts in lambs, and use it in a preliminary investigation of ACE in Norwegian sheep farms.

## 2 Materials and methods

### 2.1 Study design

#### 2.1.1 Inclusion criteria

Norwegian sheep farms (n = 80) were selected based on a previous questionnaire study performed in October 2015 (Odden et al., 2017). The inclusion criteria were: a) treatment with anticoccidials annually for at least four years, b) coccidiosis-related symptoms in lambs treated with an anticoccidial, and c) flock size of more than 60 winter-fed ewes. The geographical location of the farms was consistent with the population density of sheep farms in Norway (Supplementary data 1) (Statistics Norway, 2017). All 80 farmers, of whom 60 agreed to participate, were contacted via telephone during the winter of 2016. The 60 participating farmers received a detailed written sampling and treatment protocol, a 10 ml syringe for oral drenching, envelopes with pre-paid postage, and a “faecal spoon” to facilitate sampling of young lambs (Supplementary data 2). Farms with <5 lambs per treatment group were excluded.

#### 2.1.2 Timing of treatment and sampling

Most Norwegian ewes are winter housed, with indoor lambing in the spring i.e. March–May (Vatn, 2009). Turnout to spring pastures commonly occurs two to three weeks postpartum (Domke et al., 2011). Clinical signs due to coccidiosis are mainly seen at around turnout. Lambs may become infected before turnout, mainly due to oocyst excretion from older, already infected lambs (Taylor, 1995), or immediately after turnout, as the oocysts survive overwintering on permanent pastures (Helle, 1970; Gjerde and Helle, 1991). Current Norwegian recommendations against ovine coccidiosis consist of a single metaphylactic treatment with toltrazuril, either at turnout or around one week after turnout (Animalia, 2017).

**Fig. 1:**
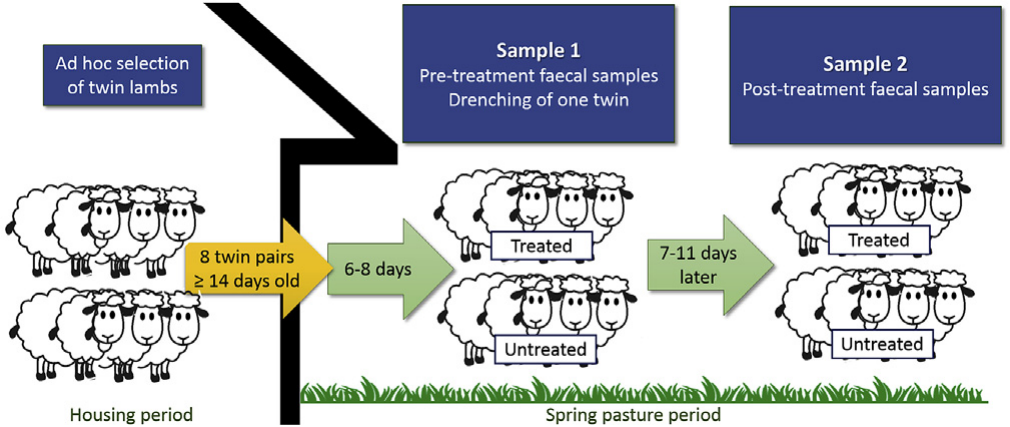
Study design. Twin lambs (n = 16 per farm) from 36 Norwegian farms with signs of coccidiosis in previous grazing seasons were included in the study. The treatment group was treated with toltrazuril (20 mg/kg) whereas the control group was left untreated. Oocyst counts were based on McMaster analysis.

Farmers enrolled in the study were instructed to identify 8 pairs of twin lambs from which they would twice collect faecal samples during the 2016 spring grazing season. Age at turnout was ≥14 days for all lambs included in the study. Sample 1 was taken 6–8 days after turnout (Fig. 1). The aims were: 1) to have a common sampling protocol for all lambs, regardless of whether they became infected indoors or after turnout, and 2) to collect the first sample while oocyst excretion was either below the limit of detection, or low. At the same time as collection of the first sample, the lamb with the lowest ear tag number from each twin pair was treated with 20 mg/kg toltrazuril (Baycox^®^ Sheep vet, Bayer Animal Health). Farmers were encouraged to weigh the lambs prior to treatment. Sample 2 was collected from the same animals 7–11 days after Sample 1; that is, before any oocysts ingested post-treatment could have resulted in additional oocyst excretion. All faecal samples were collected per rectum from individual lambs using a “faecal spoon”.

#### 2.1.3 Evaluation of faecal samples

The faecal consistency was scored on a scale from one to five (Holm et al., 2014). The faecal samples were stored for a maximum of 7 days at 4 °C and the rate of oocyst excretion was determined using a modified McMaster technique with a theoretical sensitivity of 5 oocysts per gram (OPG). Briefly, water was added to 1–4 g of faeces, which was homogenised, filtered, concentrated by centrifugation and mixed with flotation fluid (saturated sodium chloride with glucose; density: 1.27 g/ml; sample/flotation fluid ratio: 1:1 to 1:2 depending on volume of sediment). A subsample (0.6 ml) was then transferred to a disposable counting chamber fitted with a thin coverglass, which facilitated detection, and the oocysts were enumerated at 200/400× magnification. In samples with few oocysts (OPG < 10,000) the whole chamber was evaluated, whereas one row (≈1/20) or three fields of vision (≈1/200) of the chamber was counted in samples with higher numbers of oocysts (Henriksen and Aagaard, 1976; Henriksen and Korsholm, 1984). One hundred *Eimeria* oocysts from all samples with OPG ≥1000 were examined by light microscopy at 400× magnification. The oocysts were identified to species level without sporulation, using morphological criteria (Eckert et al., 1995).

### 2.2 Evaluation of anticoccidial efficacy

#### 2.2.1 Statistical justification

**Fig. 2:**
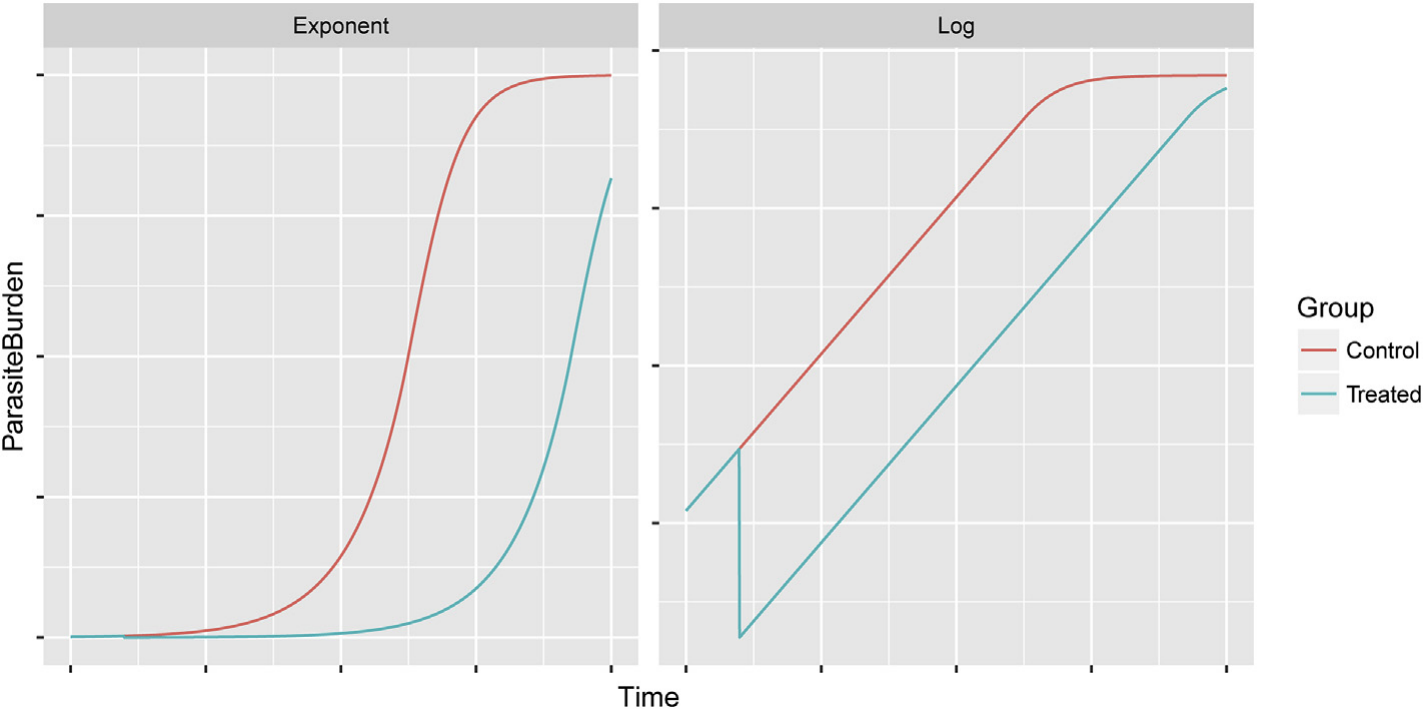
Graphical illustration of the theoretical relationship between an exponentially increasing true parasite burden on the exponent scale (left) and the log of the same quantity (right) over time. A proportional reduction in the simulated ‘treated’ (blue) mean occurring 1/10 into the time span results in the log of the two quantities increasing in parallel during the exponential phase of the control mean (red). This exponential phase ends around 6/10 into the time span, after which a plateau can be seen on the exponent scale and the lines cease to be parallel on the log scale. (For interpretation of the references to colour in this figure legend, the reader is referred to the Web version of this article.)

Previously published observations of oocyst excretion in lambs have shown that excretion follows an exponential pattern initially, followed by a plateau phase (Chapman, 1974; Gregory et al., 1989). This implies that the logarithm of the expectation of oocyst excretion increases linearly during the initial phase, followed by a reduction in the rate of increase during the subsequent plateau phase (Fig. 2). This known relationship has the useful feature that an anticoccidial intervention at any time point has no effect on the slope of the linear relationship with time, so that anticoccidial efficacy can be calculated as the absolute difference in log expectation of oocyst count at any point during the exponential growth phase. However, this assumes that parasite replication also follows an exponential rise between the time of treatment and the time of oocyst quantification, and therefore that the plateau phase of oocyst excretion has not yet been reached. Thus, three conditions must be satisfied regarding timing of anticoccidial treatment and FOCRT quantification:

1. Treatment must be given after the initial infection of the lambs.
2. A minimum time must be allowed between treatment and FOCRT to allow a reduction in oocyst shedding to become detectable.
3. Both treatment and the FOCRT must be performed during the phase of exponential rise in excretion.

Additionally, although not a strict requirement for the procedure to be valid, greater statistical confidence will be obtained in the mean count estimates, and therefore FOCRT percentage, if the mean oocyst counts are relatively high (Denwood et al., 2012). Therefore, the theoretical optimal time for the FOCRT (Sample 2) to be conducted, from a statistical point of view, is as close as possible to the end of the phase of exponential rise in excretion. We note that this statistical consideration is somewhat at odds with the optimal time point for most effective control of the parasite, for which an earlier treatment is likely to be preferable.

#### 2.2.2 Identifying the phase of exponential rise in excretion rate

There are currently no guidelines for identifying when oocyst excretion in the field is in the phase of exponential increase. We therefore followed a simple heuristic, based on oocyst counts derived from the untreated control animals at the time of the pre-treatment and post-treatment sampling. This heuristic assumes that the dynamics of parasite replication during the exponential phase are similar between farms, which is a substantially simplifying assumption, given that there are multiple species of *Eimeria* with different cycle parameters within the host animal, and that different farms may have different species.

The heuristic used was as follows. Firstly, a crude estimation of the linear effect of days between oocyst observations on the mean of the logarithmically transformed oocyst counts in the untreated animals at each farm was made (referred to as the “slope”). For this procedure, a fixed constant of 1 was added to all data before transformation to avoid numerical problems with observations of zero. Secondly, the results for all farms were compared graphically in order to identify a qualitatively appropriate threshold above which the estimated slopes were subjectively judged to be consistent with those obtained from farms with the greatest change in mean log faecal oocyst count (FOC), and therefore assumed to be representing the exponential phase. A second, lower threshold was also identified above which the slopes were deemed to be only moderately consistent with those obtained from farms with the greatest change in FOC. Data from farms for which the lower threshold for slope was not met were not included in the analyses due to the apparently poor timing of sampling and treatment.

#### 2.2.3 Analysis of faecal oocyst count reduction

The farms for which the data from the control animals were judged to indicate sampling had been performed during the exponential phase were analysed for faecal oocyst count reduction (FOCR) using a generalised linear mixed model, implemented using the lme4 package (Bates et al., 2015) for R (R Core Team, 2017). Only the post-treatment counts from treated and control animals were used for evaluation of efficacy due to the theoretical justification of the linear increase in log FOC given in section 2.2.1, as well as empirical evidence for a lack of relationship between the pre-treatment mean and these efficacy estimates (data not shown). A Poisson distribution with log link was used as the response distribution for the number of oocysts, using an offset within the model to take into account the dilution factor applied during counting of each sample. Random effects of observation and twin pair were used to describe the extra-Poisson variation (over dispersion) within the data, and all farms were analysed separately. Confidence intervals (CIs) for the geometric mean FOCR were obtained using parametric bootstrapping with 500 iterations. The FOCRT results then enabled classification of ACE at the farm level, based on 95% CI, as “good efficacy” (lower 95% CI > 90%), “reduced efficacy” (upper 95% CI < 95%), or inconclusive (neither of the above is true), based on equivalent classifications for arithmetic mean reductions as previously used by Geurden et al. (2015) and Peña-Espinoza et al. (2016) in investigations of anthelmintic efficiency.

## 3 Results

### 3.1 Identifying the exponential growth phase

**Fig. 3:**
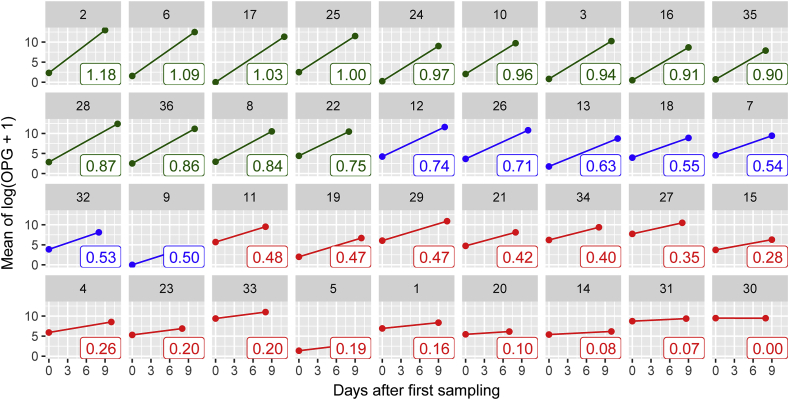
Identification of the exponential growth phase of the oocyst excretion. Graphs illustrate the slope between the mean log OPG and time (pre- and post-treatment samples) for the control lambs in the 36 farms complying with the sampling protocol. Classification was performed with **confidence** in farms with a slope of ≥0.75 (green), and with **caution** if the slope was ≥0.5 and < 0.75 (blue). Data from farms for which these thresholds were not met were deemed **unclassifiabl**e due to the poor timing of sampling (red). The number at the bottom right of each panel indicates the estimated slope for the given flock (panel title). (For interpretation of the references to colour in this figure legend, the reader is referred to the Web version of this article.)

Of the 60 farms that initially agreed to participate in the study, 49 completed the sampling. However, 13 farms were excluded due to lack of compliance with the sampling protocol, leaving 36 farms for which the increase in mean log OPG could be assessed in the control animals. Based on these estimates, along with the assumption that the parasite dynamics (and therefore exponential rate of increase) should be similar between farms for which sampling was during the exponential phase (see comments regarding this assumption in section 2.2.2), a threshold of approximately 0.75 was identified. Above this threshold, the absolute increase in mean log OPG in untreated lambs seemed to be sufficiently consistent for us to be confident that both treatment and sampling were conducted at appropriate time points for the analyses (Fig. 3). A further threshold of 0.5, was identified above which the timing is broadly consistent with the required increase in OPG in untreated lambs, but also where the possibility of sub-optimal timing cannot be excluded. Farms for which the increase in control lambs was below 0.5 were deemed to represent farms where the timing of sampling and treatment were not during the phase of exponential increase. Based on these criteria, 13 farms were considered to have been sampled at the appropriate time for our purposes and could therefore provide a useful estimate of drug efficacy, and 7 farms were deemed to have possibly been sampled at a suboptimal time and could therefore yield a drug efficacy estimate that should be analysed with caution. The data from the remaining 16 farms were considered unsuitable for further analysis as treatment and/or sampling were not during the exponential phase of oocyst excretion.

### 3.2 Assessment of efficacy

The 20 farms for which treatment and sampling were deemed to have either been consistent with, or broadly consistent with, appropriate timing were then analysed for ACE. Of the 13 farms classified with confidence, 7 were found to have good ACE, 4 were inconclusive, and 2 had reduced treatment efficacy (Table 1). Of the 7 farms classified with caution, 6 had good ACE and 1 was inconclusive. Mean and median OPG from all the 36 farms complying with the sampling protocol can be found in Table 2, and the oocyst counts from all included lambs can be found in Supplementary data 3.

**Table 1:** Maximum likelihood estimates and 95% confidence intervals (CI) for the geometric mean efficacy based on post-treatment oocyst counts for the 20 classifiable sheep farms. The slope gives the change in mean log OPG per day in the controls, and was used to determine if drug efficacy could be calculated: 13 of the flocks could be evaluated with confidence (slope ≥0.75) and 7 of the flocks were evaluated with caution (0.5 ≤ slope < 0.75).

Farm	*n*		Slope	Mean efficacy (%)	Lower 95% CI	Higher 95% CI	Interpretation
	Control	Treated					
10^a^	8	8	0.96	37.8	−58.3	73.3	Reduced efficacy
22^a^	8	8	0.75	81.7	53.1	94.0	Reduced efficacy
35^a^	7	7	0.90	56.0	−433.9	96.6	Inconclusive
16^a^	7	6	0.91	81.3	−103.8	98.9	Inconclusive
6^a^	7	8	1.09	96.0	72.7	99.3	Inconclusive
28^a^	6	6	0.87	95.4	86.5	98.4	Inconclusive
8	8	8	0.84	100.0	99.7	100.0	Efficacious
3	8	8	0.94	99.3	95.0	99.9	Efficacious
17^a^	6	8	1.03	99.3	93.6	99.9	Efficacious
2	8	8	1.18	99.5	96.8	99.9	Efficacious
25^a^	7	7	1.00	99.5	96.6	99.9	Efficacious
24^a^	7	8	0.97	99.8	98.6	100.0	Efficacious
36^a^	8	8	0.86	99.8	98.4	100.0	Efficacious
9^a^	8	8	0.50	97.6	16.5	100.0	Caution: inconclusive
13^a^	8	8	0.63	100.0	100.0	100.0	Caution: efficacious
26^a^	8	8	0.71	100.0	100.0	100.0	Caution: efficacious
7	8	8	0.54	99.4	97.3	99.8	Caution: efficacious
12^a^	8	8	0.74	99.5	96.4	99.9	Caution: efficacious
32^a^	8	8	0.53	99.8	99.2	100.0	Caution: efficacious
18^a^	6	6	0.55	99.9	99.0	100.0	Caution: efficacious

a Farms from which body weights at treatment were available.

**Table 2:** Oocyst counts pre- and post-treatment in toltrazuril treated lambs and untreated controls from the 36 farms complying with the sampling protocol. Arithmetic mean: A-mean; geometric mean: G-mean and median oocysts per gram (OPG).

Farm	Sample 1						Sample 2					
	Treated			Control			Treated			Control		
	A-mean	G-mean	Median	A-mean	G-mean	Median	A-mean	G-mean	Median	A-mean	G-mean	Median
1	1691.9	454.5	665.0	3993.1	998.6	2795.0	742.5	96.8	65.0	20373.1	4269.1	4840.0
2	5.6	12.3	0.0	175.0	220.3	0.0	31719.4	2289.1	1537.5	796962.5	434112.9	649500.0
3	6903.1	874.3	0.0	6.3	25.0	0.0	3545.0	347.6	217.5	120957.5	28274.0	38600.0
4	1178.8	580.5	20.0	33903.1	12810.4	1712.5	979.4	715.3	360.0	34691.3	4998.2	15850.0
5	40000.0	240000.0	0.0	9.5	13.8	2.5	23031.7	736.6	105.0	140.8	69.6	17.5
6	78461.4	11530.7	0.0	8.8	10.8	5.0	63010.6	10770.7	19050.0	394271.4	268180.9	364000.0
7	6991.3	473.5	370.0	141.9	92.5	34450.3	260.6	206.5	110.0	16668.1	12340.6	13100.0
8	340.6	51.4	0.0	802.5	343.6	2.5	386.3	83.6	5.0	46440.0	36473.6	58600.0
9	0.0		0.0	0.0		0.0	9.4	14.6	5.0	1831.3	186.5	10.0
10	36.9	31.5	10.0	4283.1	217.2	0.0	88583.1	10338.4	11577.5	36303.8	16651.7	21700.0
11	138855.6	293.0	195.0	13166.3	657.4	51312.5	323.8	172.9	167.5	18421.3	13427.8	14700.0
12	64060.0	689.9	5.0	19786.3	265.6	17.5	10748.8	548.8	795.0	167975.0	108179.3	165000.0
13	83828.1	5739.0	5.0	66.9	108.9	0.0	1.9	7.1	0.0	309127.5	6087.3	9880.0
14	21357.5	991.3	3065.0	18635.6	217.1	42.5	3.1	10.0	0.0	17991.9	3726.0	472.5
15	524.4	148.3	5.0	595.6	67.1	25.0	3.1	10.0	0.0	11368.6	1470.2	8695.0
16	0.0		0.0	4.3	30.0	0.0	10642.5	1072.7	1820.0	29298.6	5818.4	12800.0
17	0.0		0.0	0.0		0.0	3638.8	550.0	842.5	240960.8	83343.9	276000.0
18	42008.0	18822.7	23000.0	1012.5	374.6	2805.0	55.0	28.0	5.0	33958.3	7179.3	12550.0
19	80.0	640.0	0.0	18.8	13.1	7.5	1.4	10.0	0.0	4541.9	792.9	757.5
20	128262.5	278198.1	0.0	54519.4	54073.8	4527.5	762.5	731.8	0.0	29780.7	1267.2	2445.0
21	102764.4	6311.4	320.0	6687.9	742.5	175.0	129.4	79.9	32.5	93361.3	3297.5	7567.5
22	1915.0	110.5	7.5	8001.9	354.2	50.0	19973.8	6340.0	4885.0	57913.1	34616.6	50000.0
23	96601.3	4230.8	40.0	43156.9	1116.2	25.0	28.1	21.9	0.0	22013.1	2583.0	6232.5
24	0.0		0.0	0.7	5.0	0.0	886.3	126.6	7.5	22245.0	8182.9	17600.0
25	2117.1	270.5	10.0	1693.6	74.6	5.0	2784.3	517.2	190.0	256910.0	99538.7	221200.0
26	7186.9	1681.0	8715.0	10192.5	1482.7	47.5	9.4	18.7	0.0	225352.5	48547.0	27950.0
27	24008.8	12488.0	915.0	6543.1	2258.3	22107.5	999.4	696.2	225.0	200101.3	35781.5	32800.0
28	7295.0	374.3	15.0	260.8	69.5	15.0	24566.7	10982.1	8100.0	284233.3	240255.5	239500.0
29	85386.3	416.7	330870.0	16203.8	3131.5	52300.0	25531.4	17417.5	20400.0	103425.0	53751.7	40500.0
30	208383.1	84689.6	6100.0	171998.8	51253.1	27950.0	2828.8	783.1	697.5	27607.5	12939.1	25925.0
31	32433.8	2448.7	114242.5	106513.8	6197.6	702.5	6787.9	1829.5	897.5	77640.0	11681.4	23900.0
32	432.1	42.2	5.0	11560.6	74.8	5.0	11.3	12.0	10.0	7839.4	3298.0	5547.5
33	54625.0	11426.8	8172.5	42547.5	11990.0	17300.0	3573.8	1399.3	2097.5	141297.5	59903.4	91300.0
34	18411.9	1065.2	1370.0	8442.5	481.2	422.5	10470.0	249.4	85.0	27636.9	11697.0	9930.0
35	1.4	10.0	0.0	20.0	140.0	0.0	22897.1	1257.8	495.0	52500.7	9852.9	10100.0
36	6.9	18.2	0.0	4286.9	811.3	0.0	3581.3	220.7	112.5	163162.5	69687.7	84800.0

Of the 20 farms included in the assessment of drug efficacy, lamb weights at treatment were submitted for 16 farms (Table 1). The post-treatment composition of *Eimeria* spp. in treated lambs from the farms where reduced efficacy was detected was: 73.7% *E. faurei*, 15.5% *E. ovinoidalis*, and 10.8% other *Eimeria* spp. (farm 10), and 39.1% *E. parva*, 35.4% *E. ovinoidalis*, and 25.5% other *Eimeria* spp. (farm 22).

## 4 Discussion

Appropriate field tests are necessary in order to determine ACE and to detect potential resistance to treatment among ovine *Eimeria* isolates. However, suitable approaches for identifying ACR in farmed ruminants have not previously been developed (Joachim et al., 2018). The current work presents one approach for field evaluation of ACE against ovine *Eimeria* spp. using a method based on the WAAVP recommended FECRT for identifying resistance to anthelmintics (Coles et al., 1992), but modified to enable evaluation of drug efficacy against *Eimeria* spp., or resistance of *Eimeria* spp. against specific treatments.

One important finding of our study was that timing of anticoccidial treatment was often sub-optimal, being detected in 16 of 36 farms. Such timing could potentially result in false conclusions regarding lack of drug efficacy, as well as not providing optimal protection against clinical coccidiosis for the individual lambs.

A second important finding was an apparent reduction in the efficacy of toltrazuril against *Eimeria* spp., including the pathogenic *E. ovinoidalis,* in 2 of 20 farms for which treatment and sampling time was appropriate. It should be noted that inclusion criteria for the study included flock size, continuous use of anticoccidials for several years, and occurrence of previous episodes of diarrhoea. These are all factors that are recognised as being correlated with increased risk of ACR in poultry (Chapman et al., 2010; Peek and Landman, 2011; Lan et al., 2017). Thus, these “potential ACR farms” are not representative of Norwegian sheep farms, and further studies are needed to establish the true prevalence of ACR in Norway. However, due to the widespread dependence of sheep farmers on chemoprophylaxis to control ovine coccidiosis, the emergence of reduced efficacy of toltrazuril indicated here may have severe consequences for the sheep industry, particularly in Northern Europe, due to the limited treatment alternatives (Felleskatalogen, 2017; Läkemedelsverket, 2017; Veterinærmedicinsk Industriforening, 2017).

The main objectives of anticoccidial treatment are: 1) to decrease oocyst excretion, 2) to reduce the severity of clinical signs, and 3) to allow development of protective immunity (Taylor et al., 2011). Metaphylactic treatment (i.e., treatment administered during the prepatent period before oocyst excretion can be detected and prior to development of clinical signs) is therefore preferable. Due to the exponential oocyst excretion curve (Chapman, 1974; Gregory et al., 1989), the effect of treatment can only be assessed during the exponential growth phase of the parasite, which necessitates inclusion of both pre-treatment samples and untreated controls in the analyses. Identification of this growth phase in our study was based on evaluation of the “slope” (change in mean log OPG per day in the controls) for each farm that was used to determine if drug efficacy could be interpreted with confidence. However, this evaluation was based on thresholds chosen somewhat arbitrarily due to characteristics of our dataset, and may require adjustment following acquisition of data from more farms in future studies. Timing of treatment, and also timing of the FOCRT itself, which is supposed to be approximately one-week post-treatment, is thus a major challenge and should be based on knowledge of previous outbreaks of coccidiosis and farm management factors, such as duration of the lambing season, time of turnout, grazing conditions (±permanent pasture), and weather conditions. Historically, treatment practices in Norway relied on the assumption that lambs were infected after turnout, with the development of clinical symptoms 2–3 weeks later (Helle, 1970; Gjerde and Helle, 1986). However, results from the present study indicate that lambs in some farms are infected with *Eimeria* spp. shortly after lambing, while they are still housed indoors. This seems to be common, particularly in cases where turnout is delayed due to adverse weather conditions (unpublished results from our group). For the present study, we standardised the sampling protocol based on general knowledge of management procedures and treatment routines in Norwegian sheep farms (Odden et al., 2017). However, sampling based on specific knowledge of transmission dynamics in the individual farms would most likely have helped for at least some of the 44% of farms for which treatment timing was sub-optimal.

Another factor that is known to be associated with accelerating the development of drug resistance is under-dosing with the drug (Smith et al., 1999; Wolstenholme et al., 2004). In our study, the farmers were responsible for treating their animals with the correct dosage. The farmers were encouraged to weigh their lambs prior to treatment, and a dosage table and a syringe were provided to enable accurate dosages being used. Although the accuracy of dosing was not investigated, lambs from all farms where reduced efficacy or inconclusive results were found had been weighed prior to treatment. Therefore, although under-dosing cannot be excluded, it does not seem to be a likely cause of the reduced drug efficacy.

There has been extensive discussion of the statistical aspects of interpreting the data from FECRT, including the relative merits of arithmetic and geometric means. Miller et al. (2006) concluded that the results of a FECRT based on an arithmetic mean reduction in egg counts can be inconsistent, whereas Dobson et al. (2009) suggested that arithmetic means provide better estimates of parasite egg output than geometric means. However, the situation regarding the data from *Eimeria* FOCRT is quite different to that for nematode FECRT in that the distribution of oocyst counts is typically far more skewed than that of egg counts, with occasional extremely high oocyst count observations. This is partially a reflection of the multiple rounds of intracellular asexual reproduction in the *Eimeria* lifecycle, and that do not occur with nematodes. In this situation of highly over-dispersed parasite populations with inconsistent variation, the geometric mean is a more appropriate estimator of the central tendency parameter (Smothers et al., 1999). More fundamentally, the statistical justification presented in section 2.2.1 suggests that considering the change in arithmetic mean of the data on the log scale is the most appropriate course of action, and this is equivalent to considering the change in geometric mean on the exponent scale. We therefore believe that considering the geometric mean reduction for treated animals is most appropriate. This also facilitates the use of a more standard frequentist modelling approach using an over-dispersed Poisson distribution with log link and fixed effect of time interval to estimate the change in geometric mean count, with CIs generated using a relatively standard parametric bootstrapping procedure.

Other widely discussed statistical aspects of interpreting data from FECRT relate to the sample size and the sensitivity of the laboratory method. The sensitivity of commonly used McMaster methods for performing *Eimeria* oocyst counts usually range between 5 and 50 OPG (Reeg et al., 2005; Saratsis et al., 2011). The method used in our study has a theoretical sensitivity of 5 OPG, which, given the extremely high mean OPG values, is of sufficient sensitivity to ensure the oocyst count values will be high enough to minimise the proportion of false/excess zeros that may otherwise affect the distribution of counts and bias the final results (Denwood et al., 2008; Love et al., 2017). In FECRT-calculations, diagnostic accuracy can be improved by increasing the sample size and lowering the detection limit (Levecke et al., 2012). Coles et al. (1992) suggested a minimum sample size of 15 animals per treatment group. In the present study, the sample size of 8 lambs per treatment group was chosen as a pragmatic compromise between the statistical importance of a large sample size and the number of lambs that we expected the farmers to be able to sample twice. However, based on the relatively large proportion of farms that were classified as inconclusive, we recommend that this sample size should be increased in future studies.

Previous studies investigating the effects of anticoccidials in sheep have mostly compared different drugs with respect to oocyst excretion, faecal consistency, and weight gain without calculating the efficacy of the individual drugs (Gjerde et al., 2009; Le Sueur et al., 2009; Diaferia et al., 2013). The classification thresholds used in our study were based on the figures of 90% and 95% used by Geurden et al. (2015) and Peña-Espinoza et al. (2016) in anthelmintic studies, as there is currently no published target efficacy available for toltrazuril. The same classification targets were used by de Souza Rodrigues et al. (2017) who investigated the efficacy of different toltrazuril treatment strategies in Brazil. However, we note that although these figures seem reasonable in the absence of alternatives, there is no specific evidence to support the direct translation of 90% and 95% efficacy targets from their intended context of estimating the arithmetic mean efficacy of anthelmintic compounds against nematodes to the quite different context of estimating the geometric mean efficacy of anticoccidial compounds. The lack of published target efficacy was highlighted by Joachim et al. (2018) as one of the main challenges evaluating ACE in farm animals, and accentuate the importance of performing CETs in order to diagnose ACR properly. Consequently, there is a need for additional research into the expected geometric mean efficacy of anticoccidial drugs in a ‘susceptible’ population, after which the currently used arbitrary thresholds may be modified on the basis of evidence.

The Federation of Veterinarians of Europe has recently highlighted the importance of coccidiostats being only available by veterinary prescription (FVE, 2016), which is an important measure to control their use, and thereby potentially extend their efficacious period. By applying the method described here, we were able to produce the first evidence-based description of reduced toltrazuril efficacy against ovine *Eimeria* spp., and to highlight the importance of ensuring that treatment timing is appropriate. However, the validity of our results requires confirmation by CET. The present results suggest that the threat of emerging ACR should be taken seriously in order to safeguard animal welfare and future productivity of the sheep industry. Additional studies to establish the true prevalence of ACR and the *Eimeria* spp. involved are warranted.

## Declaration of interest

None.
