# Supplementary material for: Field evaluation of anticoccidial efficacy: A novel approach demonstrates reduced efficacy of toltrazuril against ovine Eimeria spp. in Norway
Source: Int J Parasitol Drugs Drug Resist. 2018 May 16;8(2):304–11. doi: 10.1016/j.ijpddr.2018.05.002 (PMC6039322; doi:10.1016/j.ijpddr.2018.05.002)
Supplement: Multimedia component 1 [file mmc1.docx]

**Supplementary data 1**


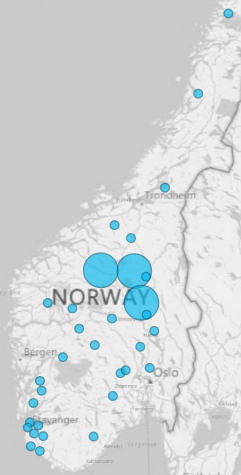


Geographical location of the 36 sheep farms included in the study. Each dot represents the municipality of the farm, and the size illustrates the number of farms per municipality (small = 1, large = 2).
