# Supplementary material for: Field evaluation of anticoccidial efficacy: A novel approach demonstrates reduced efficacy of toltrazuril against ovine Eimeria spp. in Norway
Source: Int J Parasitol Drugs Drug Resist. 2018 May 16;8(2):304–11. doi: 10.1016/j.ijpddr.2018.05.002 (PMC6039322; doi:10.1016/j.ijpddr.2018.05.002)
Supplement: Multimedia component 2 [file mmc2.docx]

**Supplementary data 2**


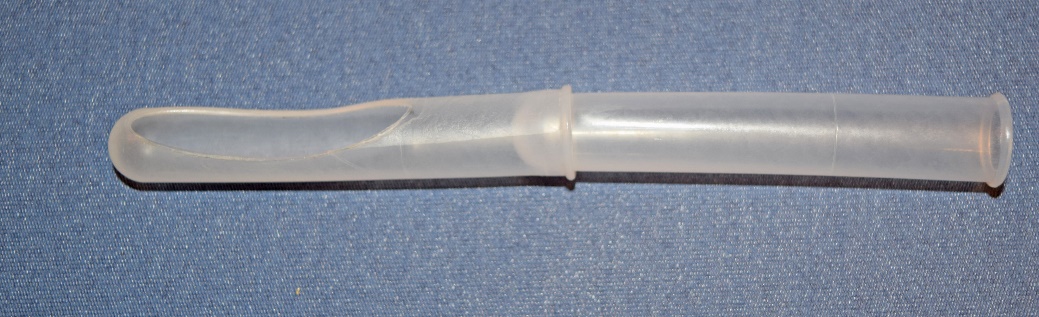

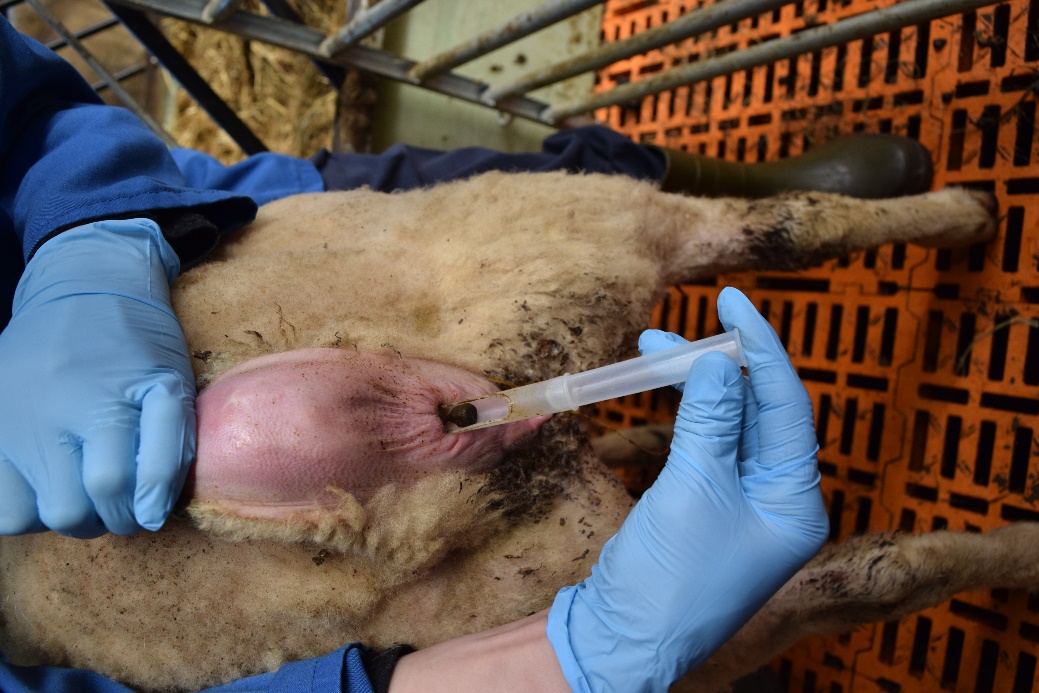


The “faecal spoon” consists of two polypropylene tubes with rounded bottoms pushed into each other. An oval part of the wall of the distal tube is removed by scalpel, and the edges are smoothened by heat to avoid damage of the rectal mucosa.
